# Prioritization of thermal energy techniques by employing picture fuzzy soft power average and geometric aggregation operators

**DOI:** 10.1038/s41598-023-27387-9

**Published:** 2023-01-30

**Authors:** Tahir Mahmood, Jabbar Ahmmad, Jeonghwan Gwak, Naeem Jan

**Affiliations:** 1 Department of Mathematics and Statistics, International Islamic University, Islamabad, Pakistan; 2 Department of Software, Korea National University of Transportation, Chungju, 27469 Korea; 3 Department of Biomedical Engineering, Korea National University of Transportation, Chungju, 27469 Korea; 4 Department of AI Robotics Engineering, Korea National University of Transportation, Chungju, 27469 Korea; 5 Department of IT & Energy Convergence (BK21 FOUR), Korea National University of Transportation, Chungju, 27469 Korea

**Keywords:** Engineering, Mathematics and computing

## Abstract

Energy storage is a way of storing energy to reduce imbalances between demand and energy production. The ability to store electricity and use it later is one of the keys to reaching large quantities of renewable energy on the grid. There are several methods to store energy such as mechanical, electrical, chemical, electrochemical, and thermal energy. Regarding their operation, storage, and cost, the choice of these energy storage techniques appears to be interesting. This issue becomes very serious when there involves uncertainty. To consider this kind of uncertain information, **a** picture fuzzy soft set is found to be a more appropriate parameterization tool to deal with imprecise data. Based on the advanced structure of picture fuzzy soft set, here in this article, firstly, we have developed the notions of basic operational laws for picture fuzzy soft numbers. Then based on these developed operational laws, we have established the notions of picture fuzzy soft power average $\left({\mathrm{PFS}}_{\mathrm{ft}}\mathrm{PA}\right)$, weighted picture fuzzy soft power average $\left({\mathrm{WPFS}}_{\mathrm{ft}}\mathrm{PA}\right)$ and ordered weighted picture fuzzy soft power average $\left({\mathrm{OWPFS}}_{\mathrm{ft}}\mathrm{PA}\right)$ aggregation operators. Moreover, we have introduced the notions for picture fuzzy soft power geometric $\left({\mathrm{PFS}}_{\mathrm{ft}}\mathrm{PG}\right)$, weighted picture fuzzy soft power geometric $\left({\mathrm{WPFS}}_{\mathrm{ft}}\mathrm{PG}\right)$ and ordered weighted picture fuzzy soft power geometric $\left({\mathrm{OWPFS}}_{\mathrm{ft}}\mathrm{PG}\right)$ aggregation operators. Furthermore, we have established the application of picture fuzzy soft power aggregation operators for the selection of thermal energy storage techniques. For this, we have developed a decision-making approach along with an explanatory example to show the effective use of the developed theory. Furthermore, a comparative analysis of the introduced work shows the advancement of developed notions.

## Introduction

Energy storage techniques help to store the energy that can be used further in the future to cover energy problems. The thermal energy storage technique (TEST) is considered to be the most crucial energy technique. Dincer^1^ proved in his research that TEST is the key energy storage technique for energy conservation. Economic reasons have an impact on energy conversion systems, and this has made TEST more prominent. Kocak et al.^2^ reveal that TEST is a useful technique and it has many applications in industry. Such TEST systems have a lot of potential for expanding the use of thermal energy equipment on an optional basis. There are typically three different types of TEST, namely, sensible TEST, latent TEST, and thermochemical TEST. The necessary storage time typically affects the choice of TEST. TESTs seem to be among the most appealing thermal applications in this area.

The fuzzy set (FS)^3^ originated by Zadeh is a great achievement for dealing with ambiguous data to reduce uncertainty. The theory of FS has been extensively used in different fields. The fuzzy TOPSIS technique was developed by Cavallaro^4^, who then used it for the evaluation of thermal energy storage in concentrating solar power projects. Gumus et al.^5^ additionally suggested fuzzy AHP and fuzzy GRA approach for selecting hydrogen energy systems. Soft set $\left({S}_{ft}S\right)$ introduced by Molodtsov^6^ is one of the value structures that use parameterization tools that can reduce uncertainty in more decent ways. The conception of ${S}_{ft}S$ has made remarkable contributions in different fields like medical^7^ and MCDM approaches^8^. Feng et al.^9^ use the idea of ${S}_{ft}S$ in three-way decision-making problems and established three-way decision-making on canonical soft sets of hesitant fuzzy sets.

Many new developments have been made in this regard and some structures have been developed like fuzzy soft set^10^ $F{S}_{ft}S$, intuitionistic fuzzy soft set $\left(IF{S}_{ft}S\right)$^11^, Pythagorean fuzzy soft set $\left(PyF{S}_{ft}S\right)$^12^ and q-rung orthopair fuzzy soft set^13^ $\left(q-ROF{S}_{ft}S\right).$ Many scholars have applied similar ideas to various fields, such as cleaner production evaluation for the aviation industry by Peng and Li^14^ using $F{S}_{ft}S. IF{S}_{ft}S$ is a more advanced structure because it covers membership grade (MG) and non-membership grade (NMG) by using the circumstances that the sum (MG, NMG) must belong to [0, 1]. $IF{S}_{ft}S$ provides more space to decision-makers and they have utilized this structure in different fields. Khan et al.^15^ use the concept of $IF{S}_{ft}S$ into the decision support system. Furthermore, based on Archimedean t-norms of $IF{S}_{ft}S$, some generalized Maclaurin symmetric mean aggregation operations have been proposed by Garg and Arora^16^. Moreover, Hooda et al.^17^ use the concept of $IF{S}_{ft}S$ to medical fields and provide its applications. Moreover, Garg and Arora^18^ introduced generalized $IF{S}_{ft}$ power aggregation operators based on generalized t-norms. Also, some new methods have been introduced like the PROMETHEE method have been introduced by Feng et al.^19^ based on $IF{S}_{ft}S$. Feng et al.^20^ proposed another view on generalized $IF{S}_{ft}S$ and discussed its applications to MADM problems.$PyF{S}_{ft}S$ uses the more advanced condition that $sum\left({MG}^{2}, {NMG}^{2}\right)$ must belong to [0, 1]. Based on this more advanced structure, an extended $PyF{S}_{ft}S$ computing strategy for systems of environmental management was proposed by Ding et al.^21^ for renewable energy pricing. Moreover, Zulqarnain et al.^22^ proposed TOPSIS methods using the environment of the correlation coefficient for $PyF{S}_{ft}S$ and provide its applications towards supply chain management. Furthermore, the applications of $PyF{S}_{ft}S$ in green supplier chain management has been developed by Zulqarnain et al.^23^. $PyF{S}_{ft}S$ is a valuable structure but in many cases when decision-makers supply $0.8$ as MG and $0.7$ as NMG then note that ${0.8}^{2}+{0.7}^{2}\notin \left[0, 1\right].$ It means that $PyF{S}_{ft}S$ is a limited structure. $q-ROF{S}_{ft}S$ can cover that issue more effectively and uses the condition that $sum \left({MG}^{q}, {NMG}^{q}\right)\in \left[0, 1\right]. q-ROF{S}_{ft}S$ is a more advanced structure and provides more space for decision-makers. Many researchers have used this notation for different applications. Zulqarnain et al.^24^ used the notion of $q-ROF{S}_{ft}S$ in aggregation operators and introduced some interactive aggregation operators. The application of Einstein aggregation operators based on $q-ROF{S}_{ft}Ss$ has been given in^25^. Hamid et al.^26^ proposed the MCDM and TOPSIS approach under the environment of $q-ROF{S}_{ft}S$. Furthermore, Chinram et al.^27^ introduced the notion of $q-ROF{S}_{ft}$ geometric aggregation operators and used these notions in decision-making approaches. Also, Abbas et al.^28^ use the conception of $q-ROF{S}_{ft}$ Bonferroni means operators to construct the decision-making study. Moreover, Zulqarnain et al.^29^ established Einstein geometric aggregation operators based on $q-ROF{S}_{ft}S$ and applied these notions to handle MCDM problems.

**Figure 1:**
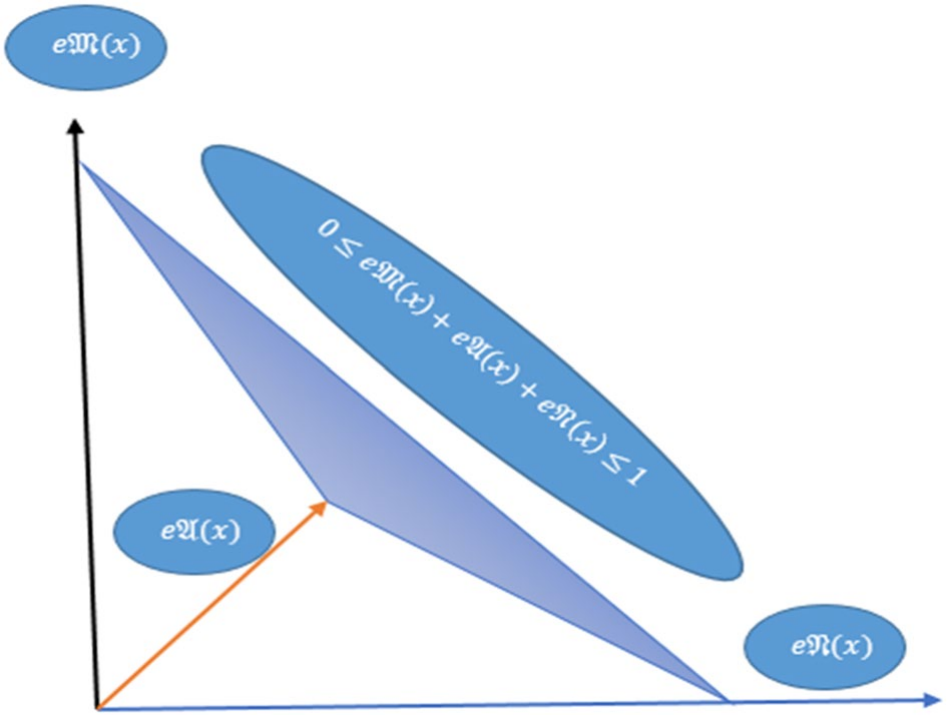
Space of all $PF{S}_{ft}Ns$.

Although the idea of a picture fuzzy set (PFS)^30^ is a more generalized structure but this structure lacks the parameterization tool. Note that all the above ideas like $F{S}_{ft}S, IF{S}_{ft}S, PyF{S}_{ft}S\,{\text{and}}\,q-ROF{S}_{ft}S$ are limited structure because when the decision maker needs to utilize the abstinence grade (AG) into its structure then all the above theories fail to cover abstinence grade. Moreover, there are some situations where multiple possible responses from human beings are required such as yes, no, abstain, and refusal. Note that only two aspects of the human opinion on an uncertain occurrence, the yes or no type symbolized by the MG and NMG, were discussed by the ideas of $IF{S}_{ft}S, PyF{S}_{ft}S\,{\text{and}}\, q-ROF{S}_{ft}S.$ Human opinion, however, is not limited to yes or no responses; it also includes abstinence grades and refusal grades. Take voting as an example, where one has the option of voting for, against, abstaining from, or refusing to cast a vote. Consequently, the picture fuzzy soft set uses three forms of grades MG, NMG, and AG with the condition that the sum (MG, NMG, AG) must belong to [0, 1]. The space of all picture fuzzy soft numbers is shown in Fig. 1. To, cover these drawbacks, Yang et al.^31^ constructed the conception of a picture fuzzy soft set $\left(PF{S}_{ft}S\right)$. $PF{S}_{ft}S$ is a parameterization structure and it can discuss the AG in its structure along with MG and NMG having the condition that the sum (MG, AG, NMG) must belong to [0, 1]. Picture fuzzy soft is a strong structure because.

1. When we ignore the AG in the structure of $PF{S}_{ft}S$, then it reduces to $IF{S}_{ft}S$.
2. If we use only one parameter then $PF{S}_{ft}S$ reduces to picture fuzzy sets.
3. Power aggregation operators were introduced by Yager^32^ and if we ignore the support element, the power average reduces to a simple average. Also, if all the support is the same then the power average reduces to a simple average. Many developments have been made on this aggregation operator like Jiang et al.^33^ use the more generalized structure of intuitionistic fuzzy set to construct the thought of power aggregation operators. Also, Pythagorean fuzzy power aggregation operators were established by Wei et al.^34^.
4. Note that if we ignore the support element and then introduced $PF{S}_{ft}PA$ aggregation operators reduce to simple picture fuzzy soft average aggregation operators. Similarly $PF{S}_{ft}PG$ aggregation operators reduce to simple picture fuzzy soft geometric aggregation operators. So it means that picture fuzzy soft average and geometric aggregation operators can be taken as special cases for these introduced picture fuzzy soft power aggregation operators.

So main contribution of this study is given by

1. To develop the generalized operational laws for picture fuzzy soft sets.
2. To introduce some picture fuzzy soft power aggregation operators like $PF{S}_{ft}PA, WPF{S}_{ft}PA, OWPF{S}_{ft}PA$ and $PF{S}_{ft}PG$, $WPF{S}_{ft}PG\,{\text{and}}\, OWPF{S}_{ft}PG$ aggregation operators.
3. To establish an algorithm to show the effective use of these gation operators for the selection of best thermal energy techniques.

Moreover, the space of picture fuzzy soft numbers is given in Fig. 1.

Based on these observations, here in this article, we have used the notion of $PF{S}_{ft}S$ and we have designed some new aggregation operators called picture fuzzy soft power average and power geometric aggregation operators. Furthermore, we have developed the characteristics of these developed notions. There are several methods to store energy such as mechanical, electrical, chemical, electrochemical, and thermal energy. Here we have developed the application of picture fuzzy soft power average and power geometric aggregation for the selection of thermal energy storage techniques. For this, we have developed a decision-making approach along with an explanatory example to show the effective use of this developed work.

The remaining text is given as: We covered some fundamental definitions of the soft set, picture fuzzy set, picture fuzzy soft set, and power aggregation operators in “Preliminaries”. The fundamental ideas of picture fuzzy soft power average aggregation operators are covered in “Picture fuzzy soft power average aggregation operators” section. We covered the concepts of picture fuzzy soft power geometric aggregation operators in “Picture fuzzy soft power geometric aggregation operators” section. To demonstrate the use of these created principles, we established the DM approach and offered an algorithm in “Decision-making approach” along with a descriptive example. The comparison of these conceptions with various existing notions is discussed in “Comparative analysis”. Conclusion remarks are covered in “Conclusion” section.

## Preliminaries

We will go through the fundamental definitions of a soft set, picture fuzzy set, picture fuzzy soft set, and power aggregation operator in this part.

### Definition 1^6^:

Let $E$ be the set of parameters, $U$ be the universal set and $A\subset E,$ then a soft set is an ordered pair $\left(F, A\right)$ where $F:A\to P\left(U\right).$

### Definition 2^30^:

For universal set $U$, a PFS is the structure of the form such that.

$$PFS=\left\{x: e\mathfrak{M}\left(x\right), e\mathfrak{A}(x), e\mathfrak{N}\left(x\right)| x\in U\right\}$$

where $e\mathfrak{M}:U\to \left[0, 1\right], e\mathfrak{N}:U\to \left[0, 1\right]$ and $e\mathfrak{A}:U\to \left[0, 1\right]$ and $e\mathfrak{M}\left(x\right), e\mathfrak{A}(x), e\mathfrak{N}\left(x\right)$ are called MG, AG, and NMG respectively by using the condition that $0\le e\mathfrak{M}\left(x\right)+e\mathfrak{A}(x)+e\mathfrak{N}\left(x\right)\le 1$.

### Definition 3^31^:

For universal set $U$, $E$ is the set of parameters, and $A\subset E,$ a $PF{S}_{ft}S$ is the pair $\left(F, A\right)$ where $F:A\to P\left(PFS\right)$ and $P\left(PFS\right)$ is the power set for PFS defined by

$${PFS}_{{P}_{j}}\left({x}_{i}\right)=\left\{{x}_{i}: \left(e\mathfrak{M}\left({x}_{i}\right), e\mathfrak{A}({x}_{i}), e\mathfrak{N}\left({x}_{i}\right)| {x}_{i}\in U\right)\right\}$$

where $e\mathfrak{M}:U\to \left[0, 1\right], e\mathfrak{A}:U\to \left[0, 1\right] \,{\text{and}}\, e\mathfrak{N}:U\to [0, 1]$ and $e\mathfrak{M}\left({x}_{i}\right), e\mathfrak{A}({x}_{i}), e\mathfrak{N}\left({x}_{i}\right)$ represent the MG, AG, and NMG respectively by using the condition that $0\le e\mathfrak{M}\left({x}_{i}\right)+e\mathfrak{A}({x}_{i})+e\mathfrak{N}\left({x}_{i}\right)\le 1.$ For the sake of simplicity, we call ${PFS}_{{P}_{j}}\left({x}_{i}\right)=\left(e\mathfrak{M}\left({x}_{i}\right), e\mathfrak{A}({x}_{i}), e\mathfrak{N}\left({x}_{i}\right)\right)$ is a picture fuzzy soft number.

### Definition 4^32^:

Let  are the attributes, then the power averaging operator is given by
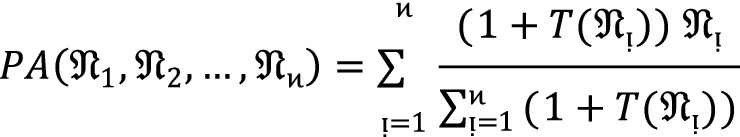
where  is the support for  form ${\mathfrak{N}}_{k},$ defined as  where  is the Hamming distance between  and ${\mathfrak{N}}_{k}$. Moreover, it satisfies the properties.

(i)
(ii)
(iii) If  then

### Example 1^32^

Assume that ${\mathfrak{N}}_{1}=2$, ${\mathfrak{N}}_{2}=4, {\mathfrak{N}}_{3}=4$ and $Sup \left(2, 4\right)=0.5, Sup \left(2, 10\right)=0.3, Sup \left(2, 11\right)=0, Sup \left(4, 10\right)=0.4, Sup \left(4, 11\right)=0.$

Now $$T\left(2\right)=Sup \left(2, 4\right)+Sup \left(2,10\right)=0.5+0.3=0.8$$

$$T\left(4\right)=Sup \left(4, 2\right)+Sup \left(\mathrm{4,10}\right)=0.5+0.4=0.9,$$

$$T\left(10\right)=Sup \left(10, 2\right)+Sup \left(\mathrm{10,4}\right)=0.3+0.4=0.7$$

And therefore

$$PA\left(2, 4, 10\right)=\frac{\left(1+0.8\right)\times 2+\left(1+0.9\right)\times 4+\left(1+0.7\right)\times 10}{\left(1+0.8\right)+\left(1+0.9\right)+\left(1+0.7\right)}=5.22$$

## Picture fuzzy soft power average aggregation operators

### Basic operational laws for picture fuzzy soft numbers

In this part of the article, we will discuss the generalized t-norm operations based on $PF{S}_{ft}Ns.$ Also, We explored the definitions of the score function and accuracy function and introduced the idea of normalized Hamming distance for $PF{S}_{ft}Ns$.

#### Definition 5:

Let $A=\langle e{\mathfrak{M}}_{A},e{\mathfrak{A}}_{A}, e{\mathfrak{N}}_{A}\rangle$, ${A}_{11}=\left({e{\mathfrak{M}}_{A}}_{11}, {e{\mathfrak{A}}_{A}}_{11}, {e{\mathfrak{N}}_{A}}_{11}\right)$ and ${A}_{12}=\left({e{\mathfrak{M}}_{A}}_{12}, {e{\mathfrak{A}}_{A}}_{12}, {e{\mathfrak{N}}_{A}}_{12}\right)$ be three $PF{S}_{ft}Ns$ and $R>0$ be any real number. Then the fundamental rules are defined by

(i)
(ii)
(iii)
(iv)

#### Example 2:

Assume that ${A}_{11}=\left(0.3 , 0.4, 0.1\right)\,{\text{and}}\,{A}_{12}=\left(0.5, 0.2, 0.3\right)\mathrm{ be\,two }\,PF{S}_{ft}Ns\,{\text{and}}\,R=2.$ Here we consider $a+b-ab$ as t-conorm and $ab$ as t-norm, then

(i) $${A}_{11}\oplus {A}_{12}=\left(\left(0.3+0.4-\left(0.3\times 0.4\right)\right), \left(0.4\times 0.2\right), \left(0.1\times 0.3\right)\right)=\left(0.65, 0.08, 0.03\right)$$
(ii) $${A}_{11}\otimes {A}_{12}=\left(\left(0.3\times 0.4\right), \left(0.4+0.3-0.4\times 0.3\right), \left(0.1+0.3-0.1\times 0.3\right)\right)=\left(0.15, 0.52, 0.37\right)$$
(iii) $$R{A}_{11}=\left(\left(1-{\left(1-0.3\right)}^{2}\right), {\left(0.4\right)}^{2}, {\left(0.1\right)}^{2}\right)=\left(0.51, 0.16, 0.01\right)$$
(iv) $${A}^{R}=\left({\left(0.3\right)}^{2}, {\left(0.4\right)}^{2}, \left(1-{\left(1-0.1\right)}^{2}\right)\right)=\left(0.09, 0.16, 0.19\right)$$

#### Definition 6:

Let  be the family of $PF{S}_{ft}Ns$, the notions of score function and accuracy function are given by


1


And
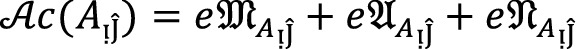
where  and .

Note that for two  and , we have

(i) if  then
(ii) if  then
(iii) if  then
  (i) if  then
  (ii) if  then
  (iii) if  then

#### Definition 7:

Let $A=\left({e{\mathfrak{M}}_{A}}_{11}, {e{\mathfrak{A}}_{A}}_{11}, {e{\mathfrak{N}}_{A}}_{11}\right) \,{\text{and}}\, B=\left({e{\mathfrak{M}}_{B}}_{11}, {e{\mathfrak{A}}_{B}}_{11}, {e{\mathfrak{N}}_{B}}_{11}\right)$ are two $PF{S}_{ft}Ns$, then the normalized Hamming distance can be defined by
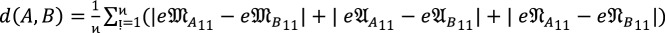


### Picture fuzzy soft power average aggregation operators

In this subsection, we have to discuss the basic definition of picture fuzzy soft power aggregation operators. Furthermore, we have to discuss the properties of these developed conceptions.

#### Definition 8:

Let  be a collection of $PF{S}_{ft}Ns$, then $PF{S}_{ft}$ power average operator is a function from  to  defined by
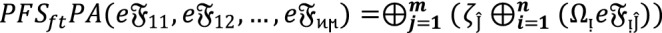
where  and  refer for support of  from .

#### Theorem 1:

Let  be the family of $PF{S}_{ft}Ns,$ then $PF{S}_{ft}PA$ aggregation operators are defined as  given by


2


#### Proof:

We apply the method of mathematical induction for  to prove this result.

## Step 1:

For , we get
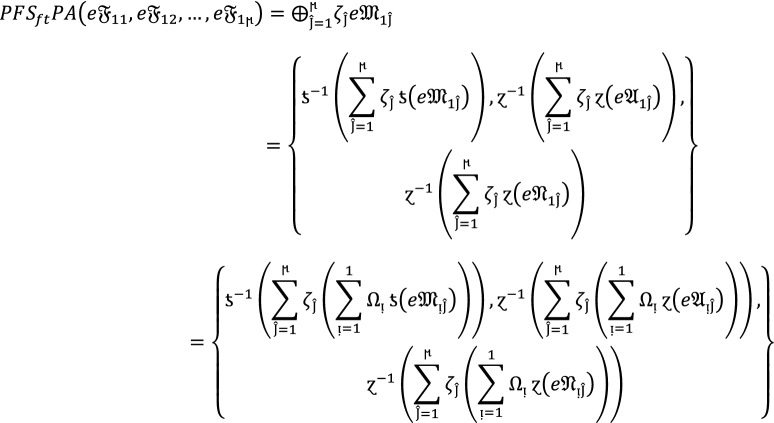


Similarly, for , we get
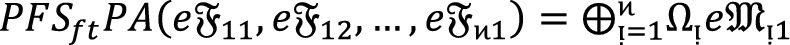



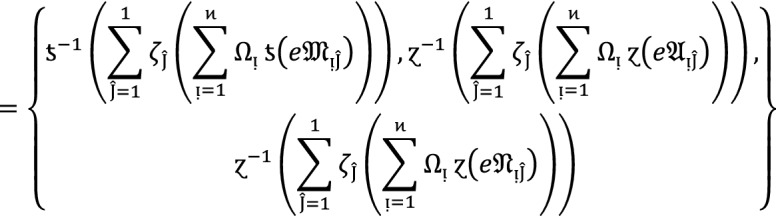


Hence result holds for  and .

## Step 2:

Now we assume that this result hold for  and  and  then for , we get
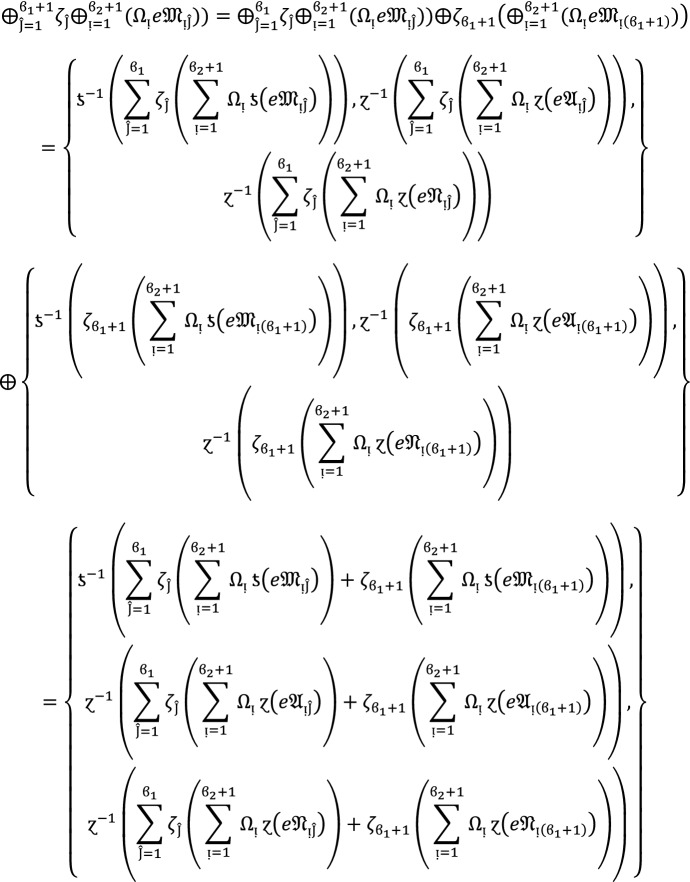



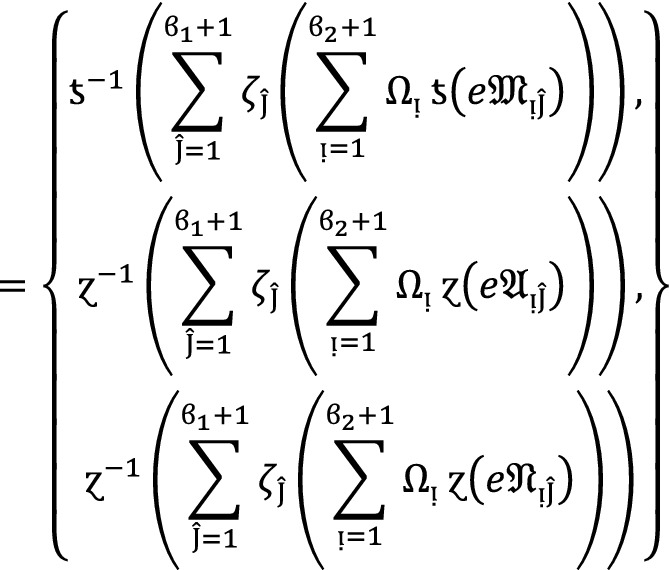


Hence result holds for  and . Hence the result is true for all positive integers .

We shall now demonstrate that the $PF{S}_{ft}PA$ aggregation operators meet the criteria listed below.

**Property 1**: (Idempotency) If  for all  then
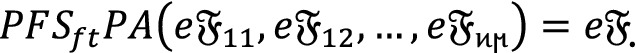


### Proof:

If  for all , then by using Eq. (2), we get
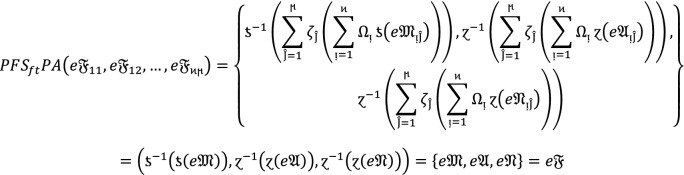


### Property 2:

If  and $e\mathfrak{F}$ are $PF{S}_{ft}Ns$, then.


3


### Proof:

Since  and $e\mathfrak{F}$ are $PF{S}_{ft}Ns$, then for all , we get
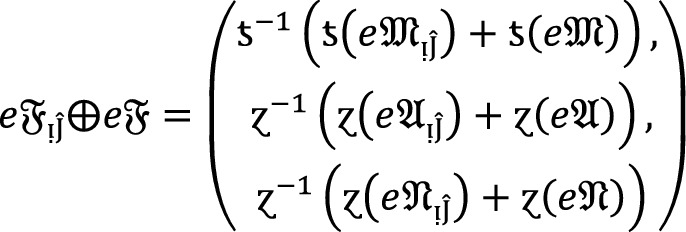


Therefore
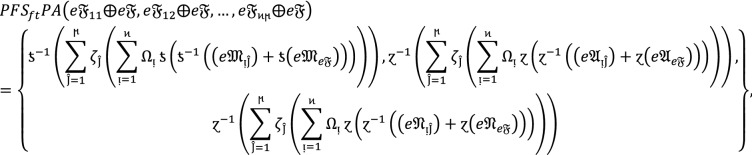



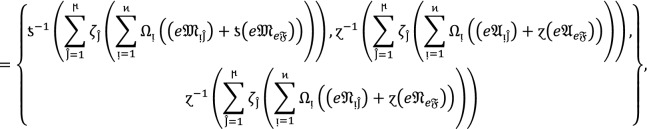



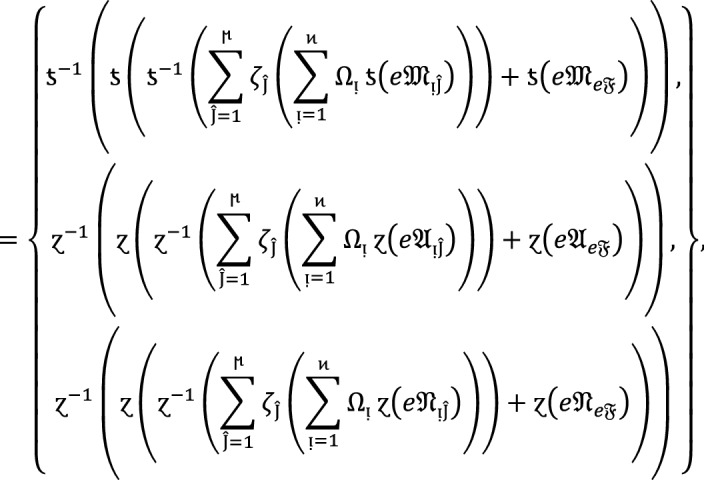



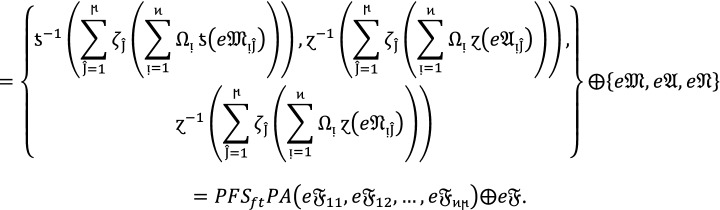


### Property 3:

For the family of $PF{S}_{ft}Ns$ and any real number ${\mathbb{R}}>0,$ we get.


4


### Proof:

If  are $PF{S}_{ft}Ns$ for  and  and ${\mathbb{R}}>0$ be a real number then
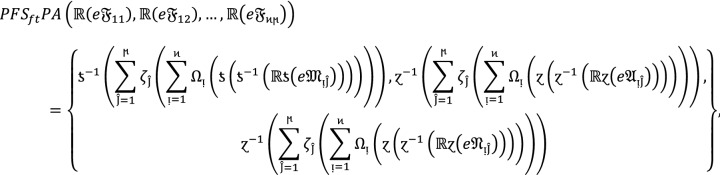



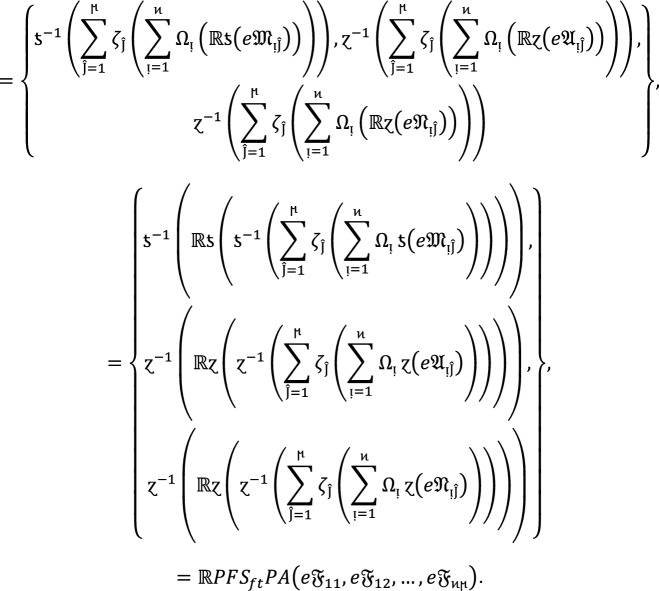


### Property 4:

If  for all  be $PF{S}_{ft}Ns$ then


5


### Proof:

For all  we have ,

Therefore,
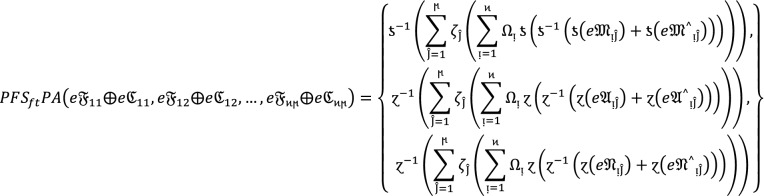



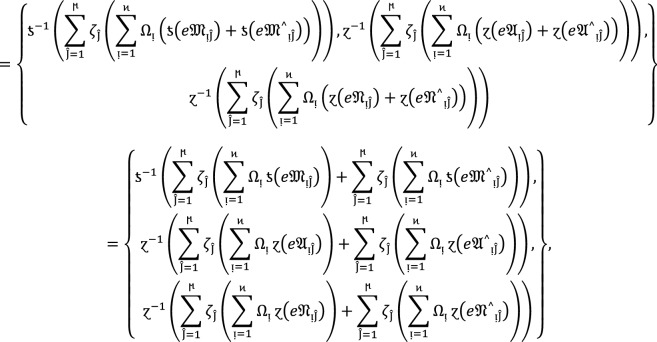



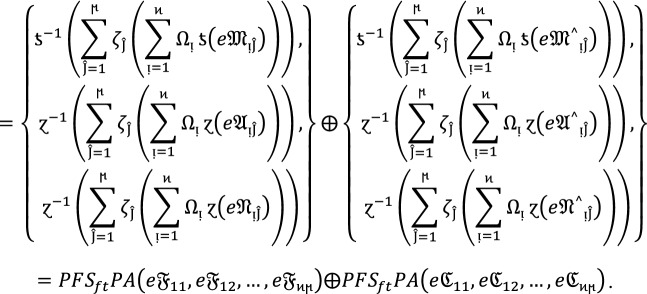


### Special cases of ${\varvec{P}}{\varvec{F}}{{\varvec{S}}}_{{\varvec{f}}{\varvec{t}}}{\varvec{P}}{\varvec{A}}$ operators

By using different values to , the initiated $PF{S}_{ft}PA$ operator degenerate as follows:

(i) If , then Eq. (2) degenerates into $PF{S}_{ft}$ Archimedean weighted average operators.
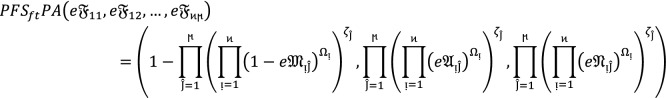
(ii) If , then Eq. (2) degenerates into $PF{S}_{ft}$ Einstein weighted average operators.
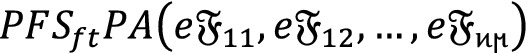

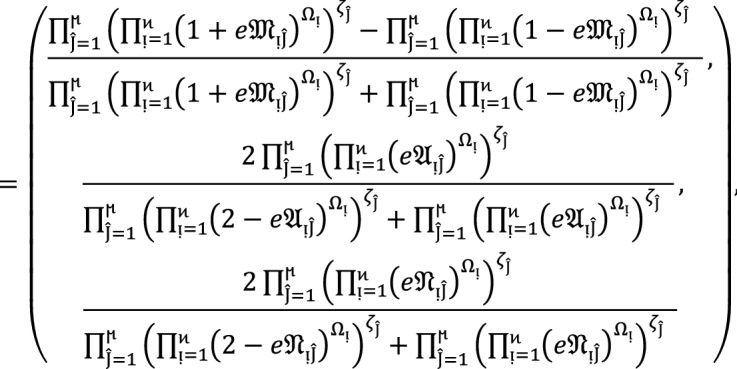
(iii) If  then Eq. (2) degenerates into $PF{S}_{ft}$ Hammer weighted average operators.
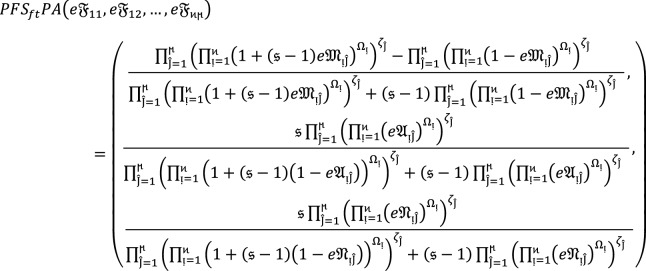

### Weighted and ordered weighted picture fuzzy soft power average aggregation operators

In this part of the article, we aim to introduce a weighted picture fuzzy soft power average $\left(WPF{S}_{ft}PA\right)$ and ordered weighted picture fuzzy soft power average $\left(OWPF{S}_{ft}PA\right)$ aggregation operators.

#### Definition 9:

Let  denote the family of $PF{S}_{ft}Ns,$ then $WPF{S}_{ft}PA$ aggregation operators are defined as  given by
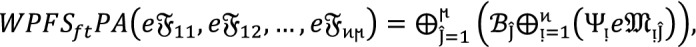
where  and the weight vectors (WVs) for the experts and parameters with the condition that  and  and .

#### Theorem 2:

Let  be the collection of $PF{S}_{ft}Ns,$ then $WPF{S}_{ft}PA$ aggregation operators are again $PF{S}_{ft}N$ given by


6


#### Proof:

Proof is similar to the proof of Theorem 1.

#### Definition 10:

Let  denote the family of $PF{S}_{ft}Ns,$ then $OWPF{S}_{ft}PA$ aggregation operators are defined as  given by
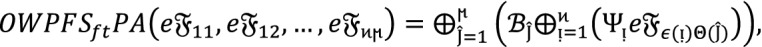
where  and  are the WVs corresponding to parameters and experts with the condition that  and . Also, $\epsilon ,\Theta$ are the permutation of  and  with a constraint that  and  for .

#### Theorem 3:

Let  denote the family of $PF{S}_{ft}Ns,$ the value obtained by $OWPF{S}_{ft}PA$ is again a $PF{S}_{ft}N$ given by


7


#### Proof:

Similar to Theorem 1, so omit here.

## Picture fuzzy soft power geometric aggregation operators

### Definition 11:

Suppose  be the collection of $PF{S}_{ft}Ns$, then $PF{S}_{ft}PG$ aggregation operators are defined as  given by.

8where  and  means the support for  from  and  is the support of  from .

### Theorem 4:

Let  denote the family of $PF{S}_{ft}Ns,$ then the value obtain by using $PF{S}_{ft}PG$ aggregation operators are again $PF{S}_{ft}N$ given as


9


### Proof:

To demonstrate this finding, we use the mathematical induction technique for .

**Step 1**

For , we get
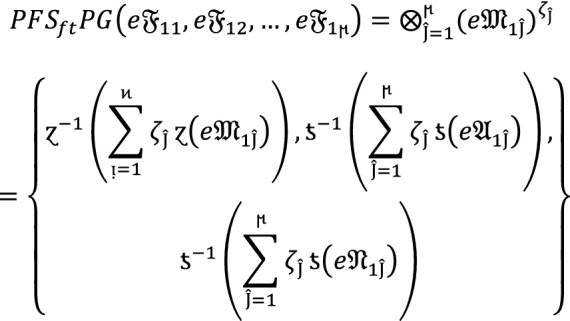



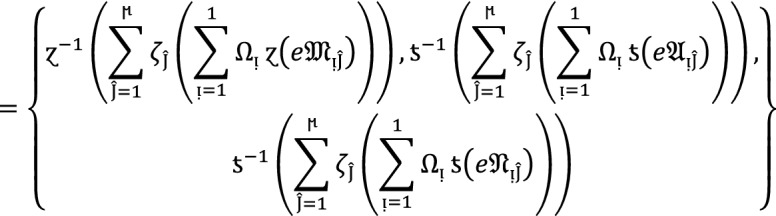


Similarly, for , we get
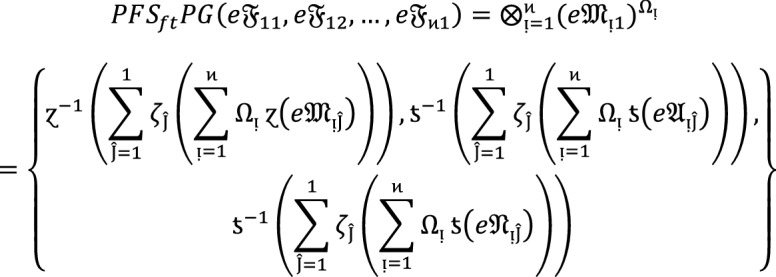


Hence result holds for  and .

**Step 2:**

Now we assume that this result hold for  and  and  then for , we get
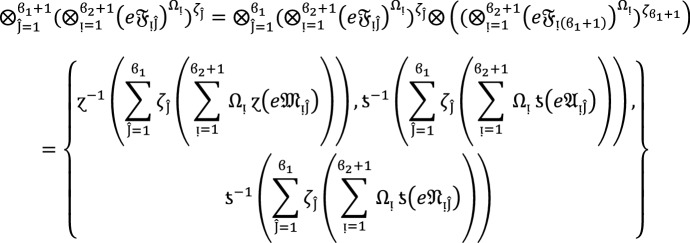



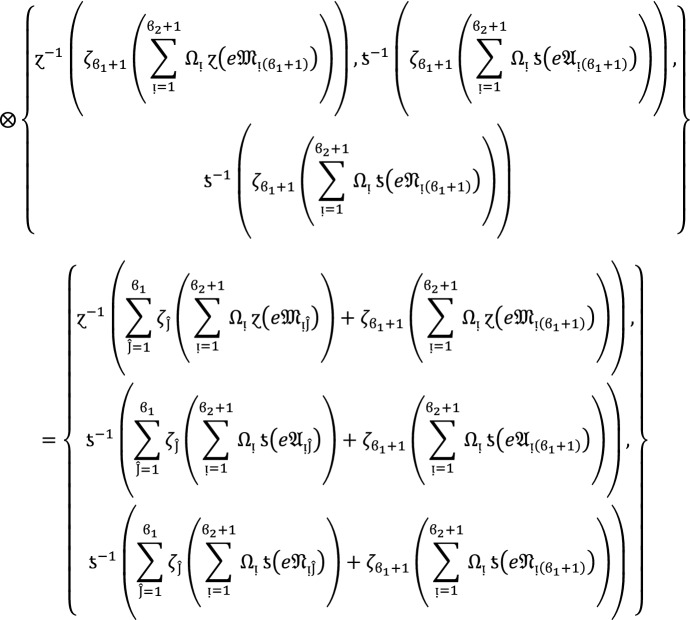



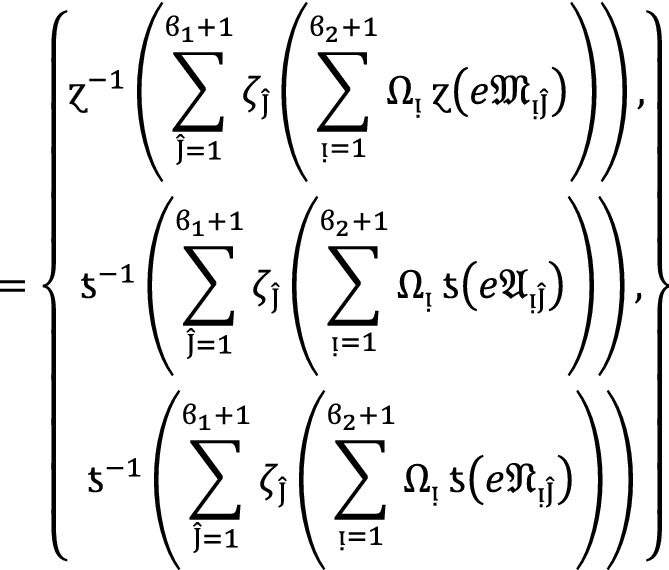


Hence the result is true for  and . Hence the result is true for all positive integers .

Now we will prove that $PF{S}_{ft}PG$ aggregation operators satisfy the following properties.

**Property 1**: (Idempotency) If  for all  then
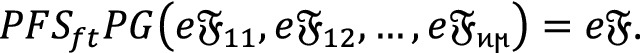


### Property 2:

If  and  are $PF{S}_{ft}Ns$, then
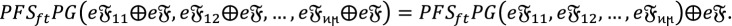


### Property 3:

For the family of $PF{S}_{ft}Ns$ and any real number ${\mathbb{R}}>0,$ we get
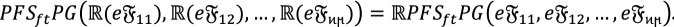


### Property 4:

If  be $PF{S}_{ft}Ns$ then.
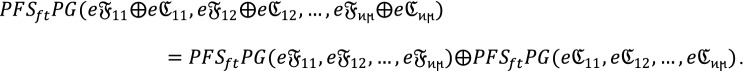


### Special cases of ${\varvec{P}}{\varvec{F}}{{\varvec{S}}}_{{\varvec{f}}{\varvec{t}}}{\varvec{P}}{\varvec{G}}$ operators

By using different values to $q,$ the initiated $PF{S}_{ft}PG$ operator degenerate as follows:

- If , then Eq. (9) degenerates into $PF{S}_{ft}$ Archimedean weighted geometric operators
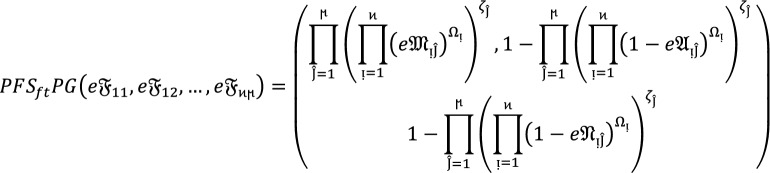

- If , then Eq. (9) degenerates into $PF{S}_{ft}$ Einstein weighted geometric operators.

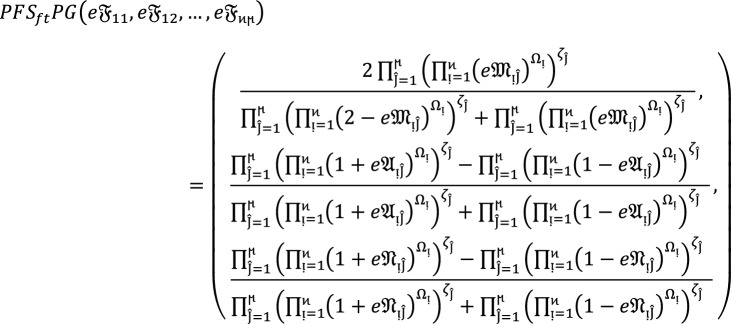

- If  then Eq. (9) degenerates into $PF{S}_{ft}$ Hammer weighted geometric operators

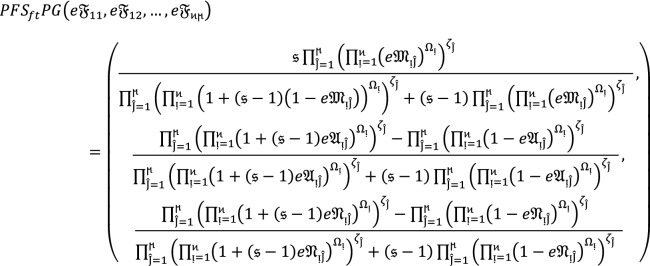


### Weighted and ordered weighted picture fuzzy soft power geometric aggregation operators

In this part of the article, we aim to introduce weighted $PF{S}_{ft}$ power geometric $\left(WPF{S}_{ft}PG\right)$ and ordered weighted $PF{S}_{ft}$ power geometric $\left(OWPF{S}_{ft}PG\right)$ aggregation operators.

#### Definition 12:

Let  be the collection of $PF{S}_{ft}Ns,$ then $WPF{S}_{ft}PG$ aggregation operators are defined as 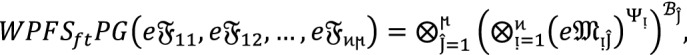
where  and  are WVs corresponding to parameters and experts with a condition that  and .

#### Theorem 5:

Let  denote the family of $PF{S}_{ft}Ns,$ then the value obtained by $WPF{S}_{ft}PG$ aggregation operators are again $PF{S}_{ft}N$ given by.


10


#### Proof:

Similar to Theorem 4.

#### Definition 13:

Let  be the family of $PF{S}_{ft}Ns,$ then $OWPF{S}_{ft}PG$ aggregation operators are defined as  given by.
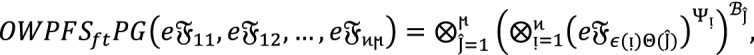
where  and  are WVs corresponding to parameters and experts with a condition that  and . Also, $\epsilon ,\Theta$ are the permutation of  and  with a constraint that  and  for .

#### Theorem 6:

Let  be the collection of $PF{S}_{ft}Ns,$ then the result obtained by $OWPF{S}_{ft}PG$ aggregation operators are again $PF{S}_{ft}N$ given by.


11


#### Proof:

Similar to Theorem 4, so we omit it here.

## Decision-making approach

### Algorithm

Let ${\mathcal{F}}^{\wr }=\left\{{\mathcal{F}}_{1}^{\wr }, {\mathcal{F}}_{2}^{\wr }, {\mathcal{F}}_{3}^{\wr },\dots , {\mathcal{F}}_{{\mathcal{Z}}^{*}}^{\wr }\right\}$ denote a set of ${\mathcal{Z}}^{*}$ alternatives,  denote the set of experts  denote the set of parameters. Assume that  are WVs corresponding to experts and parameters respectively with a condition that . Suppose analysts provide their assessment in the form of . The stepwise algorithm is given below to select supreme alternatives among the given ones.

**Step 1:** Collect the data about each alternative ${{\mathcal{F}}_{{\mathcal{Z}}^{*}}^{\wr }}^{\hslash } \left(\hslash =1, 2, 3, \dots , {\mathcal{Z}}^{*}\right)$ in the form of $PF{S}_{ft}Ns$ and summarized this data into a matrix given by
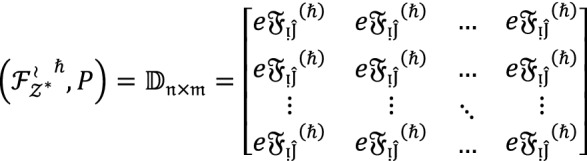


**Step 2:** Compute the support  for each expert  by using

12 where 

**Step 3:** Find out the support  by using the below formula


13


**Step 4:** Now we use $WPF{S}_{ft}PA$ or $WPF{S}_{ft}PG$ aggregation operators to aggregate the preference of different alternatives by using  into collective one ${{\mathbb{C}}^{\between }}^{\hslash }$ as follows


14


Or


15


**Step 5:** Use Definition (6) to find the score value of each alternative ${{\mathcal{F}}_{{\mathcal{Z}}^{*}}^{\wr }}^{\hslash } \left(\hslash =1, 2, 3, \dots , {\mathcal{Z}}^{*}\right).$

**Step 6:** Rank the alternatives and find the best alternative.

### Numerical example

Thermal energy is produced due to the collision of molecules and atoms as a result of a temperature rise. The concept of thermal energy is used in various fields of physics. There are three techniques for storing thermal energy: (1) Sensible TEST (2) Latent TEST (3) Thermo-chemical TEST.

#### Sensible thermal energy storage

**Figure 2:**
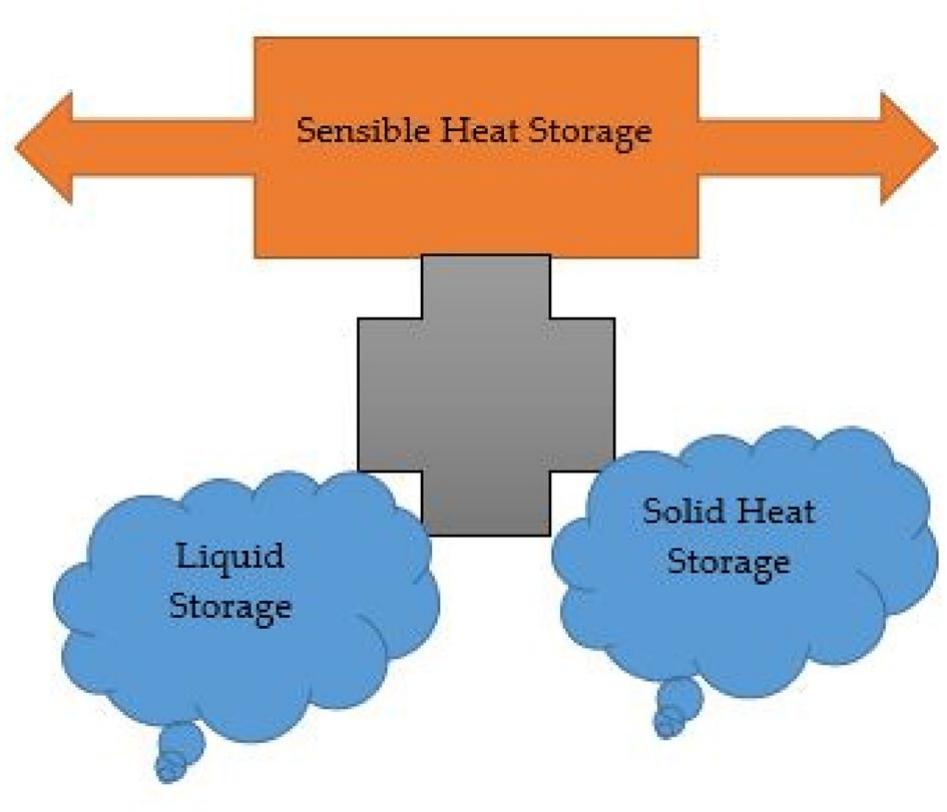
Flow chart for sensible heat storage.

Sensible heat storage works by increasing a liquid or solid's temperature to store heat and then releasing it when the temperature drops as needed. Sensible heat, which can be either a liquid or a solid, is based on an increase in the material's enthalpy from a thermodynamic perspective. The graphical presentation of the sensible heat system is given in Fig. 2.

#### Latent-heat thermal energy storage

**Figure 3:**
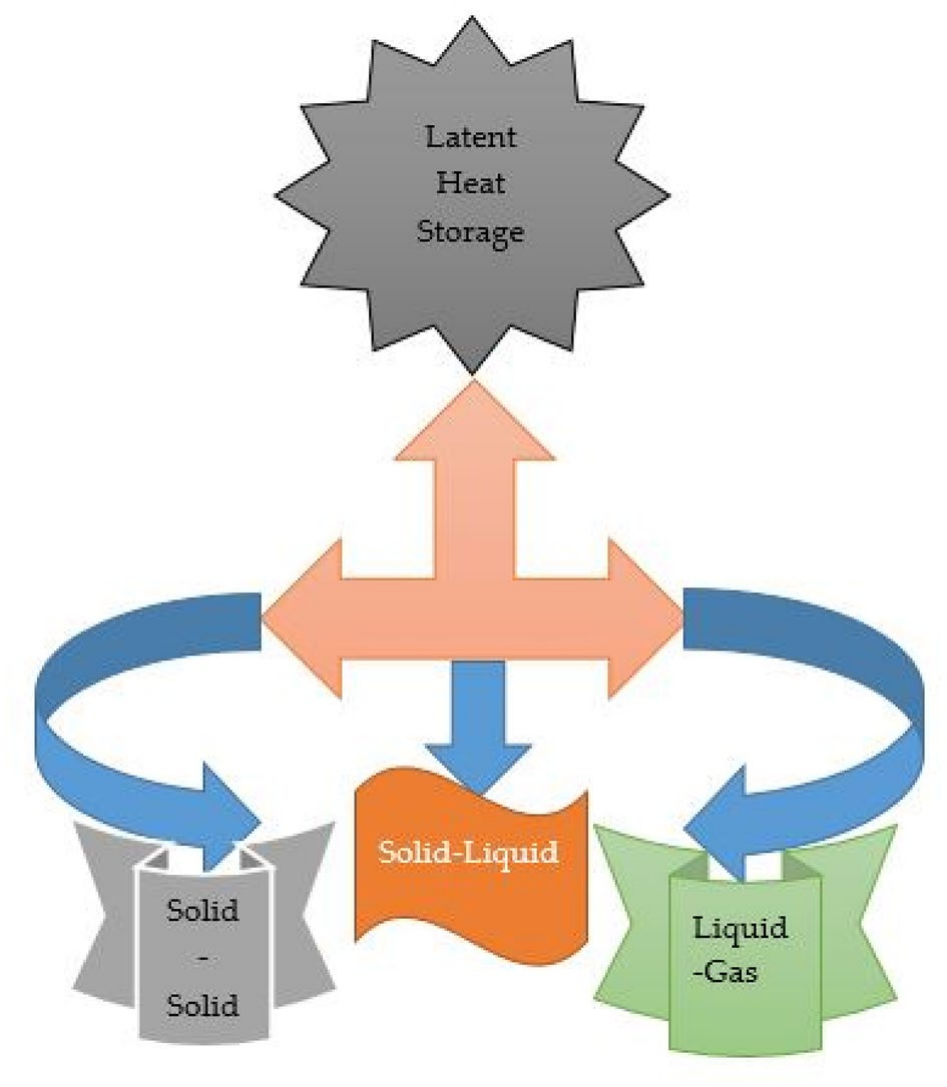
Latent heat storage.

Latent heat of storage store heat in the form of potential energy between the particles of a substance. Heat storage occurs without the storage medium significantly changing in temperature because a phase transition occurs when heat is converted from potential energy to heat in a substance. Figure 3 presents the pictorial view of the latent heat system.

#### Thermo-chemical thermal energy storage

**Figure 4:**
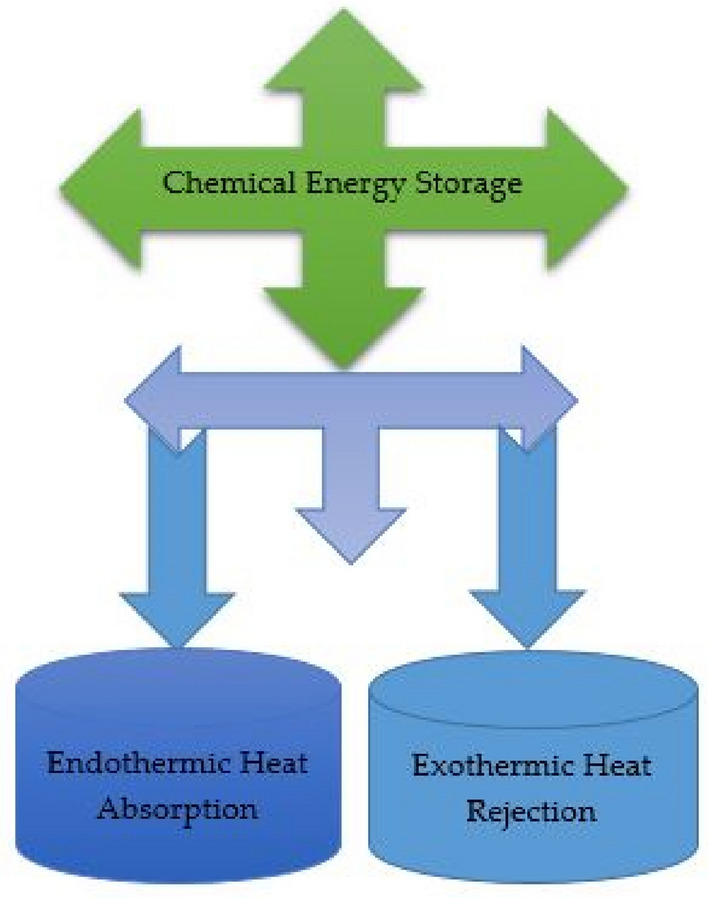
Thermo-chemical thermal energy storage technique.

Chemical energy storage is mainly constituted of batteries and renewable generated chemicals (hydrogen, fuel cells, and hydrocarbons). Here, the chemical energy of the material is used as a basis for storing and realizing thermal energy with infinite heat loss. It is an advanced thermal energy storage system and it facilitates a more efficient and clean energy system. Figure 4 represents the pictorial view of the thermo-chemical thermal energy storage system.

Our aim in this section is to propose the best thermal energy storage technologies that can further help in deciding energy departments. The overall discussion in this regard is given below.

##### Example 3:

Let ${\mathcal{F}}_{1}^{\wr }=Sensible\,thermal\,heat\,storage, {\mathcal{F}}_{2}^{\wr }=Latent\,thermal\,heat\,storage\,and\,{\mathcal{F}}_{3}^{\wr }=Thermo-chemicl\,thermal\,heat\,storage$ are three alternatives that we are going to analyze that which of the alternative is best under the five parameters ${P}_{1}^{\bowtie }=Capacity,\,{P}_{2}^{\bowtie }=Efficiency,\,{P}_{3}^{\bowtie }=Storage\,period, {P}_{4}^{\bowtie }=Charge\,and\,discharge\,time, {P}_{5}^{\bowtie }=Cost.$ Also, suppose that WVs for experts are $\left(0.11, 0.23, 0.14, 0.34, 0.18\right)$ and WVs for parameters are $\left(0.21, 0.19, 0.25, 0.17, 0.18\right)$. Now the overall discussion is given step vise.

##### By using ${\varvec{W}}{\varvec{P}}{\varvec{F}}{{\varvec{S}}}_{{\varvec{f}}{\varvec{t}}}{\varvec{P}}{\varvec{A}}$ aggregation operators

**Step 1.** Suppose a team of five experts  provide their assessment for each alternative based on proposed parameters in the form of $PF{S}_{ft}Ns$ given in Table 1, 2 and 3.

**Table 1:** Picture fuzzy soft data for ${\mathcal{F}}_{1}^{\wr }$.

	${P}_{1}^{\bowtie }$	${P}_{2}^{\bowtie }$	${P}_{3}^{\bowtie }$	${P}_{4}^{\bowtie }$	${P}_{5}^{\bowtie }$
	$\left(0.21, 0.13, 0.10\right)$	$\left(0.20, 0.22, 0.26\right)$	$\left(0.22, 0.23, 0.24\right)$	$\left(0.28, 0.30, 0.33\right)$	$\left(0.27, 0.23, 0.29\right)$
	$\left(0.22, 0.23, 0.14\right)$	$\left(0.13, 0.37, 0.26\right)$	$\left(0.18, 0.13, 0.16\right)$	$\left(0.31, 0.35, 0.24\right)$	$\left(0.20, 0.30, 0.40\right)$
	$\left(0.25, 0.16, 0.18\right)$	$\left(0.10, 0.14, 0.15\right)$	$\left(0.23, 0.11, 0.10\right)$	$\left(0.41, 0.11, 0.10\right)$	$\left(\mathrm{0.21,0} .43, 0.11\right)$
	$\left(0.11, 0.17, 0.19\right)$	$\left(0.27, 0.29, 0.25\right)$	$\left(0.11, 0.13, 0.17\right)$	$\left(0.42, 0.23, 0.27\right)$	$\left(0.12, \mathrm{0.13,0} .15\right)$
	$\left(0.41, 0.30, 0.23\right)$	$\left(0.19, 0.17, 0.15\right)$	$\left(0.51, 0.10, 0.33\right)$	$\left(0.19, 0.18, 0.16\right)$	$\left(0.16, 0.21, 0.18\right)$

**Table 2:** Picture fuzzy soft data for ${\mathcal{F}}_{2}^{\wr }$.

	${P}_{1}^{\bowtie }$	${P}_{2}^{\bowtie }$	${P}_{3}^{\bowtie }$	${P}_{4}^{\bowtie }$	${P}_{5}^{\bowtie }$
	$\left(0.12, 0.23, 0.17\right)$	$\left(0.22, 0.21, 0.23\right)$	$\left(0.12, 0.12, 0.23\right)$	$\left(0.21, 0.20, 0.30\right)$	$\left(0.12, 0.13, 0.27\right)$
	$\left(0.20, 0.21, 0.15\right)$	$\left(0.18, 0.27, 0.12\right)$	$\left(0.15, 0.16, 0.21\right)$	$\left(0.43, 0.25, 0.14\right)$	$\left(0.24, 0.20, 0.41\right)$
	$\left(0.12, 0.14, 0.16\right)$	$\left(0.17, 0.14, 0.13\right)$	$\left(0.20, 0.17, 0.15\right)$	$\left(0.28, 0.17, 0.19\right)$	$\left(0.31, 0.33, 0.34\right)$
	$\left(0.31, 0.47, 0.10\right)$	$\left(0.23, 0.49, 0.12\right)$	$\left(0.21, 0.23, 0.27\right)$	$\left(0.18, 0.19, 0.20\right)$	$\left(0.15, 0.16, 0.17\right)$
	$\left(0.41, 0.20, 0.25\right)$	$\left(0.41, 0.27, 0.10\right)$	$\left(0.41, 0.18, 0.13\right)$	$\left(0.29, 0.28, 0.36\right)$	$\left(0.18, 0.11, 0.19\right)$

**Table 3:** Picture fuzzy soft data for ${\mathcal{F}}_{3}^{\wr }$.

	${P}_{1}^{\bowtie }$	${P}_{2}^{\bowtie }$	${P}_{3}^{\bowtie }$	${P}_{4}^{\bowtie }$	${P}_{5}^{\bowtie }$
	$\left(0.15, 0.25, 0.14\right)$	$\left(0.14, 0.11, 0.13\right)$	$\left(0.17, 0.18, 0.29\right)$	$\left(0.21, 0.10, 0.20\right)$	$\left(0.15, 0.15, 0.17\right)$
	$\left(0.21, 0.24, 0.25\right)$	$\left(0.17, 0.26, 0.13\right)$	$\left(0.15, 0.36, 0.21\right)$	$\left(0.23, 0.23, 0.24\right)$	$\left(0.21, 0.22, 0.31\right)$
	$\left(0.17, 0.19, 0.13\right)$	$\left(0.14, 0.14, 0.15\right)$	$\left(0.25, 0.27, 0.15\right)$	$\left(0.21, 0.27, 0.29\right)$	$\left(0.41, 0.13, 0.24\right)$
	$\left(0.21, 0.41, 0.15\right)$	$\left(0.25, 0.46, 0.17\right)$	$\left(0.11, 0.13, 0.27\right)$	$\left(0.28, 0.15, 0.10\right)$	$\left(0.15, 0.13, 0.18\right)$
	$\left(0.31, 0.29, 0.24\right)$	$\left(0.31, 0.37, 0.30\right)$	$\left(0.21, 0.18, 0.15\right)$	$\left(0.19, 0.18, 0.16\right)$	$\left(0.13, 0.16, 0.17\right)$

**Step 2:** Compute the value of  for $\hslash =1, 2, 3$ by using Eq. (12)
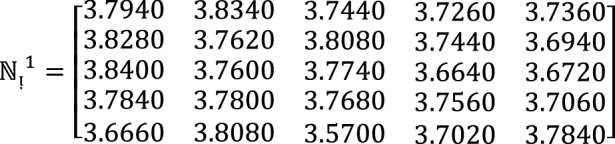



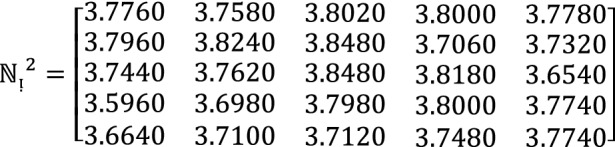



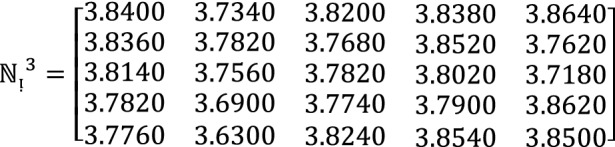


**Step 3:** Compute the value of  for $\hslash =1, 2, 3$ by using Eq. (13)
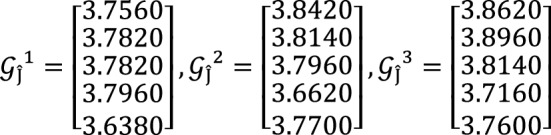


**Step 4:** By using the proposed approach of $WPF{S}_{ft}PA$ operators to aggregate different alternatives ${\mathcal{F}}^{h}\,for\,h=1, 2, 3$ by using  into collective one ${{\mathbb{C}}^{\between }}^{\hslash }$ as follows
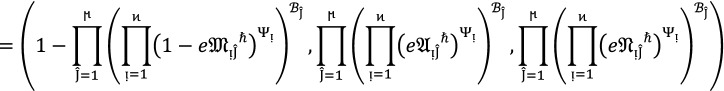


We get $${{\mathbb{C}}^{\between }}^{1}=\left(0.1502, 0.3889, 0.3821\right), {{\mathbb{C}}^{\between }}^{2}=\left(0.1455, 0.1366, 0.1249\right), {{\mathbb{C}}^{\between }}^{3}=\left(0.1266, 0.3975, 0.3777\right)$$

**Step 5:** Score values of ${{\mathbb{C}}^{\between }}^{\hslash } (h=1, 2, 3)$ are as

$$Sc\left({{\mathbb{C}}^{\between }}^{1} \right)=-0.6208, Sc\left({{\mathbb{C}}^{\between }}^{2} \right)=-0.1160, Sc\left({{\mathbb{C}}^{\between }}^{3} \right)=-0.6487$$

**Step 6:** Rank of the alternative is ${\mathcal{F}}_{2}^{\wr } >{\mathcal{F}}_{1}^{\wr } >{\mathcal{F}}_{3}^{\wr }$ and hence ${\mathcal{F}}_{2}^{\wr }$ is the best alternative.

## Comparative analysis

In this part of the article, we will discuss the comparative analysis of the developed approach with some existing notions to show the effectiveness of the proposed work. We will compare our work with Garg and Arora's^18^ method, Jiang et al.^33^ method, and Wei and Lu method^34^.

### Example 4:

A person $X$ wants to invest his income into a suitable business and he has a set of three different companies $\left\{{\mathcal{F}}_{1}^{\wr }=A\,car\,company, {\mathcal{F}}_{1}^{\wr }=A\,mobile\,company\,and {\mathcal{F}}_{1}^{\wr }=Furniture\,company\right\}$ as an alternative to investing his income. To choose the best alternative, a team of five experts is invited who assess the given alternatives based on five parameters ${P}_{1}^{\bowtie }=Risk\,analysis, {P}_{2}^{\bowtie }=Growth\,analysis, {P}_{3}^{\bowtie }=Social\,Polytical\,impact\,analysis, {P}_{4}^{\bowtie }=Environment\,imapct\,analysis\,and\,{P}_{5}^{\bowtie }=Earning\,stability.$

Let the WVs for parameters and experts are $\left(0.20, 0.21, 0.22, 0.19, 0.18\right)$ and $\left(0.13, 0.21, 0.24, 0.17, 0.25\right).$ Suppose the experts provide their assessment for each alternative in the form of $PF{S}_{ft}Ns$ as given in Table 4, 5 and 6.

**Table 4:** Picture fuzzy soft data for ${\mathcal{F}}_{1}^{\wr }$.

	${P}_{1}^{\bowtie }$	${P}_{2}^{\bowtie }$	${P}_{3}^{\bowtie }$	${P}_{4}^{\bowtie }$	${P}_{5}^{\bowtie }$
	$\left(0.20, 0.11, 0.10\right)$	$\left(0.10, 0.12, 0.25\right)$	$\left(.12, .13, .14\right)$	$\left(0.23, 0.13, 0.10\right)$	$\left(0.20, 0.24, 0.27\right)$
	$\left(0.21, 0.22, 0.11\right)$	$\left(0.23, 0.47, .16\right)$	$\left(0.17, 0.14, 0.12\right)$	$\left(0.41, 0.21, 0.27\right)$	$\left(0.21, 0.32, 0.30\right)$
	$\left(0.22, 0.14, 0.15\right)$	$\left(0.16, 0.34, 0.45\right)$	$\left(0.21, 0.15, 0.16\right)$	$\left(0.11, 0.41, .20\right)$	$\left(0.1111, 0.33, 0.21\right)$
	$\left(0.17, 0.18, 0.19\right)$	$\left(0.17, 0.21, 0.15\right)$	$\left(0.14, 0.16, 0.19\right)$	$\left(0.12, 0.13, 0.29\right)$	$\left(0.22, 0.23, 0.25\right)$
	$\left(0.31, 0.20, 0.13\right)$	$\left(0.18, 0.11, 0.12\right)$	$\left(0.31, 0.17, 0.23\right)$	$\left(0.29, 0.28, 0.26\right)$	$\left(0.26, 0.20, 0.28\right)$

**Table 5:** Picture fuzzy soft data for ${\mathcal{F}}_{2}^{\wr }$.

	${P}_{1}^{\bowtie }$	${P}_{2}^{\bowtie }$	${P}_{3}^{\bowtie }$	${P}_{4}^{\bowtie }$	${P}_{5}^{\bowtie }$
	$\left(0.11, 0.22, 0.17\right)$	$\left(0.20, 0.11, 0.13\right)$	$\left(0.17, 0.14, 0.13\right)$	$\left(0.51, 0.10, 0.11\right)$	$\left(0.17, 0.11, 0.17\right)$
	$\left(0.22, 0.11, 0.16\right)$	$\left(0.19, 0.28, 0.15\right)$	$\left(0.14, 0.26, 0.20\right)$	$\left(0.43, 0.21, 0.15\right)$	$\left(0.14, 0.23, 0.11\right)$
	$\left(0.13, 0.15, 0.17\right)$	$\left(0.19, 0.24, 0.23\right)$	$\left(0.11, 0.27, 0.25\right)$	$\left(0.18, 0.15, 0.16\right)$	$\left(0.41, 0.23, 0.14\right)$
	$\left(0.11, 0.27, 0.11\right)$	$\left(0.13, 0.19, 0.18\right)$	$\left(0.11, 0.13, 0.17\right)$	$\left(0.17, 0.18, 0.21\right)$	$\left(0.16, 0.15, 0.18\right)$
	$\left(0.21, 0.26, 0.21\right)$	$\left(0.31, 0.17, 0.19\right)$	$\left(0.21, 0.19, 0.16\right)$	$\left(0.21, 0.22, 0.16\right)$	$\left(0.19, 0.21, 0.10\right)$

**Table 6:** Picture fuzzy soft data for ${\mathcal{F}}_{3}^{\wr }$.

	${P}_{1}^{\bowtie }$	${P}_{2}^{\bowtie }$	${P}_{3}^{\bowtie }$	${P}_{4}^{\bowtie }$	${P}_{5}^{\bowtie }$
	$\left(0.13, 0.21, 0.15\right)$	$\left(0.10, 0.13, 0.15\right)$	$\left(0.14, 0.15, 0.19\right)$	$\left(0.11, 0.12, 0.21\right)$	$\left(0.15, 0.12, 0.12\right)$
	$\left(0.11, 0.20, 0.35\right)$	$\left(0.14, 0.21, 0.15\right)$	$\left(0.13, 0.31, 0.11\right)$	$\left(0.20, .22, .24\right)$	$\left(0.11, 0.12, 0.41\right)$
	$\left(0.16, 0.13, 0.23\right)$	$\left(0.11, \mathrm{0.15,0} .16\right)$	$\left(0.26, \mathrm{0.28,0} .13\right)$	$\left(0.11, 0.17, 0.19\right)$	$\left(0.21, 0.53, 0.21\right)$
	$\left(0.26, 0.21, 0.25\right)$	$\left(0.24, 0.41, 0.10\right)$	$\left(0.19, 0.23, 0.20\right)$	$\left(0.18, 0.15, 0.13\right)$	$\left(0.15, 0.16, 0.28\right)$
	$\left(0.33, \mathrm{0.25,0} .27\right)$	$\left(0.21, 0.17, 0.20\right)$	$\left(0.11, 0.17, 0.15\right)$	$\left(0.13, 0.12, 0.15\right)$	$\left(0.23, 0.26, 0.17\right)$

Now we use the weighted picture fuzzy soft power average aggregation operator for this problem to achieve the result. The overall results are given in Table 7.

**Table 7:** Overall result of the comparative study.

Methods	Score values	Ranking
Garg and Arora's^18^ method	Not applicable	Not applicable
Jiang et al. method^33^	Not applicable	Not applicable
Wei and Lu method^34^	Not applicable	Not applicable
$WPF{S}_{ft}PWA$ operator (proposed work)	$Sc\left({\mathcal{F}}_{1}^{\wr } \right)=-0.5309, Sc\left({\mathcal{F}}_{1}^{\wr } \right)=-0.0970, Sc\left({\mathcal{F}}_{1}^{\wr } \right)=-0.5585$	${\mathcal{F}}_{2}^{\wr } >{\mathcal{F}}_{1}^{\wr } >{\mathcal{F}}_{3}^{\wr }$

From the observation of the above table, we conclude that.

1. Picture fuzzy soft set is a valuable tool to tackle more advanced data. Garg and Arora's^18^ method consists of intuitionistic fuzzy soft information that uses the membership grade and non-membership grade. Although, the intuitionistic fuzzy soft set considers the parameterization tool missing the abstinence grade. Hence proposed work is dominant in existing theory.
2. Jiang et al.^33^ method consist of intuitionistic fuzzy data that is free from parameterization tool while existing notions can consider the parameterization factor in their structure. Also, intuitionistic fuzzy data used in Jiang et al.^33^ method cannot consider the abstinence grade. Note that picture fuzzy soft data used for the proposed work can solve all the above-given issues. So, the proposed approach is more effective.
3. We can see that picture fuzzy soft information provides more space to a decision maker when they need to use more advanced data in their structure.
4. Although the method proposed in Wei and Lu method^34^ consists of more generalized data of Pythagorean fuzzy sets. But we see that the Pythagorean fuzzy set also does not use the abstinence grade in its structure. Moreover, Pythagorean fuzzy data lacks the parameterization factor while the proposed work can handle both of these drawbacks. Hence in any manner, we can see that established work is more effective and superior.

## Conclusion

There exist many generalizations for fuzzy set theory but picture fuzzy soft set is a more advanced apparatus that not only cover abstinence grade but also uses the parameterization tool. Based on these observations, here we have developed the notions of aggregation operators like picture fuzzy soft power average and power geometric aggregation operators. The advantages of these developed aggregation operators are that these operators reduce to the simple form. Thus, with no support, here we can see that picture fuzzy soft power average and geometric aggregation operators reduce to simple picture fuzzy soft average and geometric aggregation operators. Moreover, when all the support is the same then picture fuzzy soft power average and geometric aggregation operators reduce to simple picture fuzzy soft average and geometric aggregation operators. Decision-making plays a vital role in all areas of life. The selection of the best technique used for the storage of thermal energy is the basic theme of these developments. We have applied the developed approach to solve decision-making problems for the selection of the best storage technique for thermal energy. We have done with comparative analysis of the introduced work to show its reliability.

The existing notion is also limited because when the decision maker comes up with $0.3$ as MG, $0.5$ as AG, and $0.4$ as NMG then the existing notion fails to handle such kind of data because the main condition of the picture fuzzy soft set has been violated in this case. Moreover, we can see that the spherical fuzzy soft structure is a more advanced structure that can handle the above-given situation because it uses more advanced conditions that $sum \left({MG}^{2}, {AG}^{2}, {NMG}^{2}\right)\in \left[0, 1\right]$. Also, the developed aggregation operator cannot handle the T-spherical fuzzy soft information because T-spherical fuzzy soft uses the data in the form of MG, AG and NMG provided that $sum \left({MG}^{q}, {AG}^{q}, {NMG}^{q}\right)\in \left[0, 1\right]$ where $q\ge 1$. Note that $PF{S}_{ft}S$ is also limited because when decision-makers come up with interval-valued picture soft numbers as given in^35^, then this structure fails to handle that kind of information.

So, in the future, we can extend this notion to the spherical fuzzy soft rough environment as given in^36^. We can extend these notions to T-spherical fuzzy sets^37,38^ and bipolar complex fuzzy sets given in^39^. We can apply this research to some novel hypotheses, such as improving digital innovation for the long-term transformation of the manufacturing sector, as suggested in^40^. We can extend these notions to neutrosophic soft sets proposed in^41^. Moreover, some new developments can be made and the idea can be extended to neutrosophic notions as established by Peng et al.^42^. Based on these introduced operational laws some new notions can be established as introduced in^43^.

In this study, the abbreviations of all ideas which are used in this manuscript are discussed in the form of Table 8. Moreover, a characteristic analysis of the introduced work with some existing notions is given in Table 9.

**Table 8:** Expressions of the abbreviations in this manuscript.

Abbreviations	Complete Name
TEST	Thermal energy storage technique
$PF{S}_{ft}S$	Picture fuzzy soft set
$PF{S}_{ft}PA$ operators	Picture fuzzy soft power average aggregation operator
$WPF{S}_{ft}PA$ operators	Weighted picture fuzzy soft power average aggregation operator
$OWPF{S}_{ft}PA$ operators	Ordered weighted picture fuzzy soft power average aggregation operator
$PF{S}_{ft}PG$ operators	Picture fuzzy soft power geometric aggregation operator
$WPF{S}_{ft}PG$ operators	Weighted picture fuzzy soft power geometric aggregation operator
$OWPF{S}_{ft}PG$ operators	Ordered weighted picture fuzzy soft power geometric aggregation operator

**Table 9:** Characteristic evaluation of different methods.

Methods	Fuzzy data	Aggregate parameter data
Garg and Arora’s Method^16^	Yes	Yes
Jiang et al. method^26^	Yes	No
Wei and Lu method^27^	Yes	No
$PF{S}_{ft}PA$ operatros	Yes	Yes
$WPF{S}_{ft}PA$ operatros	Yes	Yes
$OWPF{S}_{ft}PA$ operatros	Yes	Yes
$PF{S}_{ft}PG$ operatros	Yes	Yes
$WPF{S}_{ft}PG$ operatros	Yes	Yes

## Data Availability

All data generated or analysed during this study are included in this published article.
